# The effect of traditional Chinese medicine on psychological conditions among elderly patients with cancer: a scoping review

**DOI:** 10.1111/psyg.13182

**Published:** 2024-08-29

**Authors:** Renchuan Zhang, Pei Shi, Ying Chou, Wei Liu, Chunyu Zhang

**Affiliations:** ^1^ Infection Control Department The First Affiliated Hospital of Heilongjiang University of Chinese Medicine Harbin China; ^2^ Oncology Department The First Affiliated Hospital of Heilongjiang University of Chinese Medicine Harbin China; ^3^ Endoscopic Diagnosis and Treatment Department The First Affiliated Hospital of Heilongjiang University of Chinese Medicine Harbin China; ^4^ The Second Orthopaedics Department The First Affiliated Hospital of Heilongjiang University of Chinese Medicine Harbin China; ^5^ Nursing Department The First Affiliated Hospital of Heilongjiang University of Chinese Medicine Harbin China

**Keywords:** cancer, elderly, psychological conditions, traditional Chinese medicine

## Abstract

Coping with cancer presents a multitude of challenges that encompass every aspect of a patient's life. These challenges not only strain the body but also weigh heavily on the mind, often culminating in profound psychological distress for cancer patients. The cumulative burden of these experiences can heighten the risk of developing psychiatric disorders, exacerbating the already daunting landscape of cancer care. Therefore, this study reviewed the available research with the aim of investigating the effects of traditional Chinese medicine on psychological conditions in elderly cancer patients. In this scoping review, we applied specific criteria to select studies that focused on elderly patients with cancer. We performed an extensive search across electronic databases, including Embase, Science Direct, PubMed, Google Scholar, Scopus, Cochrane Library and Web of Science. In our investigation, we identified a total of 3870 articles related to the topic under review. Following a meticulous screening process that involved evaluating titles, abstracts, and full texts, we ultimately selected five articles deemed relevant for inclusion in this review. Among these articles, three were randomised studies, while the remaining two were review articles. The outcomes of our analysis revealed that herbal decoctions, nutritional counselling, Tai Chi and acupressure, can effectively improve various psychological outcomes in elderly cancer patients. These interventions reduce fatigue, depression, anxiety, and stress, while also enhancing sleep quality and overall mental health. The present study highlights the importance of traditional Chinese medicine in addressing the needs of elderly patients with cancer. As a result, it is recommended that further extensive research be conducted to comprehensively assess the efficacy and safety of traditional Chinese medicine in managing cancer in the elderly.

## INTRODUCTION

Cancer is a rapidly increasing chronic health condition worldwide.^1^ The most common types of cancer with high incidence rates are lung, breast, prostate, and colorectal cancers. In terms of mortality, lung, colorectal, stomach, and liver cancers cause the greatest number of deaths.^2^, ^3^ The rise in cancer cases can be attributed to factors such as metabolic comorbidities, longer lifespans, and lifestyle choices.^4^ Older adults bear a significant burden, as more than 50% of cancer diagnoses occur in those aged 65 and above.^5^ Cancer is a leading cause of mortality in this population.^4^ Dealing with cancer entails various challenges, including diagnosis, treatment side effects, sleep disturbances, coping difficulties, emotional distress, and family issues.^6^, ^7^ Such experiences can lead to significant psychological stress and increase the risk of psychiatric disorders among cancer patients.^4^ Recognising the impact of psychological well‐being on cancer care, national organisations like the National Institutes of Health (NIH), the Institute of Medicine (IOM), the National Comprehensive Cancer Network (NCCN), and the National Institute for Health and Care Excellence (NICE) recommend routine screening for psychological disorders as part of supportive and palliative cancer care.^8^

The treatment of psychological disorders in cancer patients can be divided into pharmacological and non‐pharmacological interventions. While both types have shown effectiveness, determining the first‐line recommendations based on current evidence is challenging.^9^, ^10^ Pharmacological interventions can lead to high relapse rates and various side effects like nausea, headaches, somnolence, dry mouth, and male sexual dysfunction.^11^ Additionally, there are potential harmful interactions between these drugs and cancer chemotherapeutic agents and anti‐emetics.^8^, ^12^ Psychological interventions include specific therapies such as cognitive behavioural therapy, interpersonal therapy (IPT), and behavioural activation.^13^ However, limitations such as the need for skilled providers, time commitment, high costs, and patient participation and motivation hinder the widespread application of these interventions.^8^ Non‐pharmacological interventions can complement conventional treatments and enhance quality of life for cancer patients.^14^

Complementary and alternative medicine (CAM), which is an alternative to mainstream health care, is widely used worldwide as an integral part of the medical system. It encompasses a range of treatments rooted in diverse histories and cultures.^15^ According to the World Health Organization, CAM has been utilised for centuries in maintaining health, preventing diseases, and providing treatment.^16^ It encompasses a wealth of knowledge, skills, and practices for promoting physical and mental well‐being, as well as preventing, diagnosing, improving, and treating illnesses.^17^ One prominent form of CAM is traditional Chinese medicine (TCM), which globally enjoys widespread usage.^15^ TCM has been practised for 1000s of years in China, with a focus on disease prevention and treatment.^18^ It is based on unique views on life, fitness, diseases, and approaches to preventing and treating illnesses developed over its extensive history of absorption and innovation.^19^

One of the foundational concepts in TCM is the principle of Yin and Yang. The well‐known Yin/Yang symbol depicts opposing yet complementary characteristics and functions. Yin and Yang are interdependent, with one unable to exist without the other. When Yin reaches its extreme, it transforms into Yang, and vice versa. The symbol illustrates this with a small dark circle (Yin) within the larger white area (Yang) above, and a small white circle (Yang) within the larger dark area (Yin) below. The primary objective of TCM is to achieve balance between Yin and Yang, harmonising the body, mind, and spirit. This internal equilibrium is facilitated through various means, including the use of healing foods, herbs, acupuncture, moxibustion, Tui Na massage, cupping, meditation, and exercises like Taichi and Qigong.^15^, ^20^, ^21^ In China, TCM is not considered an alternative to Western medicine, but rather an integrative complement to it.^22^ Both TCM and Western medicine hold equal importance within China's medical system.^15^ Valid evidence has confirmed the efficacy of TCM therapies in addressing psychological disorders in cancer patients.^14^, ^23^, ^24^, ^25^ Additionally, studies conducted on older individuals with cancer have indicated a reduction in psychological problems within this population.^26^, ^27^, ^28^ However, determining the optimal approach remains unclear.

Therefore, there is a crucial need for a comprehensive comparative analysis to examine the effectiveness, safety, patient preferences, and potential synergies of TCM interventions in addressing psychological disorders in cancer patients. This research would deepen our understanding of TCM's role in cancer care, and provide insights into personalised treatment approaches that align with patient needs and preferences. By bridging this gap in knowledge, this research can inform clinical practice, enhance patient‐centred care, and ultimately improve the quality of life for older cancer patients facing psychological challenges. Therefore, this scoping review aims to investigate the effect of TCM on psychological conditions among elderly patients with cancer.

## MATERIALS AND METHODS

The Preferred Reporting Items for Systematic Reviews and Meta‐Analyses 2020 approach for Scoping Review (PRISMA‐ScR) was used to conduct this study.^29^ This scoping review was conducted in five stages: (i) formulating the research question; (ii) searching for and extracting relevant studies; (iii) selecting related studies; (iv) tabulating, summarising, and synthesising the information and data; and (v) reporting the results.^30^ Following the formulation of the research question, which focused on the role of TCM in managing psychological conditions among older patients with cancer, a search strategy was developed, inclusion criteria for the selected studies were established, data extraction forms were prepared, and a data analysis program was specified.

### Information sources and searches

In order to achieve the best search strategy, researchers conducted searches across several databases, including Embase, Science Direct, PubMed, Google Scholar, Scopus, Cochrane Library and Web of Science. They used a range of keywords such as ‘stress’, ‘anxiety’, ‘depression’, ‘Post‐traumatic stress disorder’, ‘resilience’, ‘mental disorder’, ‘psychological disorder’, ‘psychological issues’, ‘sleep wake disorders’, ‘dyssomnias’, ‘fatigue’, ‘insomnia’, ‘traditional Chinese medicine’, ‘Yin/Yang’, ‘Chinese decoction’, ‘elderly’, ‘cancer’, ‘carcinoma’, ‘neoplasm’, and ‘tumour’.

Researchers used Marshall's study^21^ to identify various types of TCM. In this study, the terms ‘Chinese herbal medicine’, ‘acupuncture’, ‘moxibustion’, ‘acupressure’, ‘Tui Na massage’, ‘cupping’, ‘nutrition and lifestyle consultation’, ‘meditation’, ‘Taichi and Qigong’ are all categorised as TCM (see Appendix I).

### Inclusion and exclusion criteria

The inclusion criteria included all experimental, quasi‐experimental, systematic and meta‐analysis, clinical trial, review, interventional, observational and *in vitro* articles, appearing as full‐text articles published in reputable journals. Additionally, qualitative, and mix‐method studies were considered. The exclusion criteria covered articles that did not specifically investigate the impact of TCM on psychological issues among the elderly with cancer, as well as editorial, letter, conference and protocol articles and studies published in languages other than English.

### Selection of relevant studies

**Figure 1:**
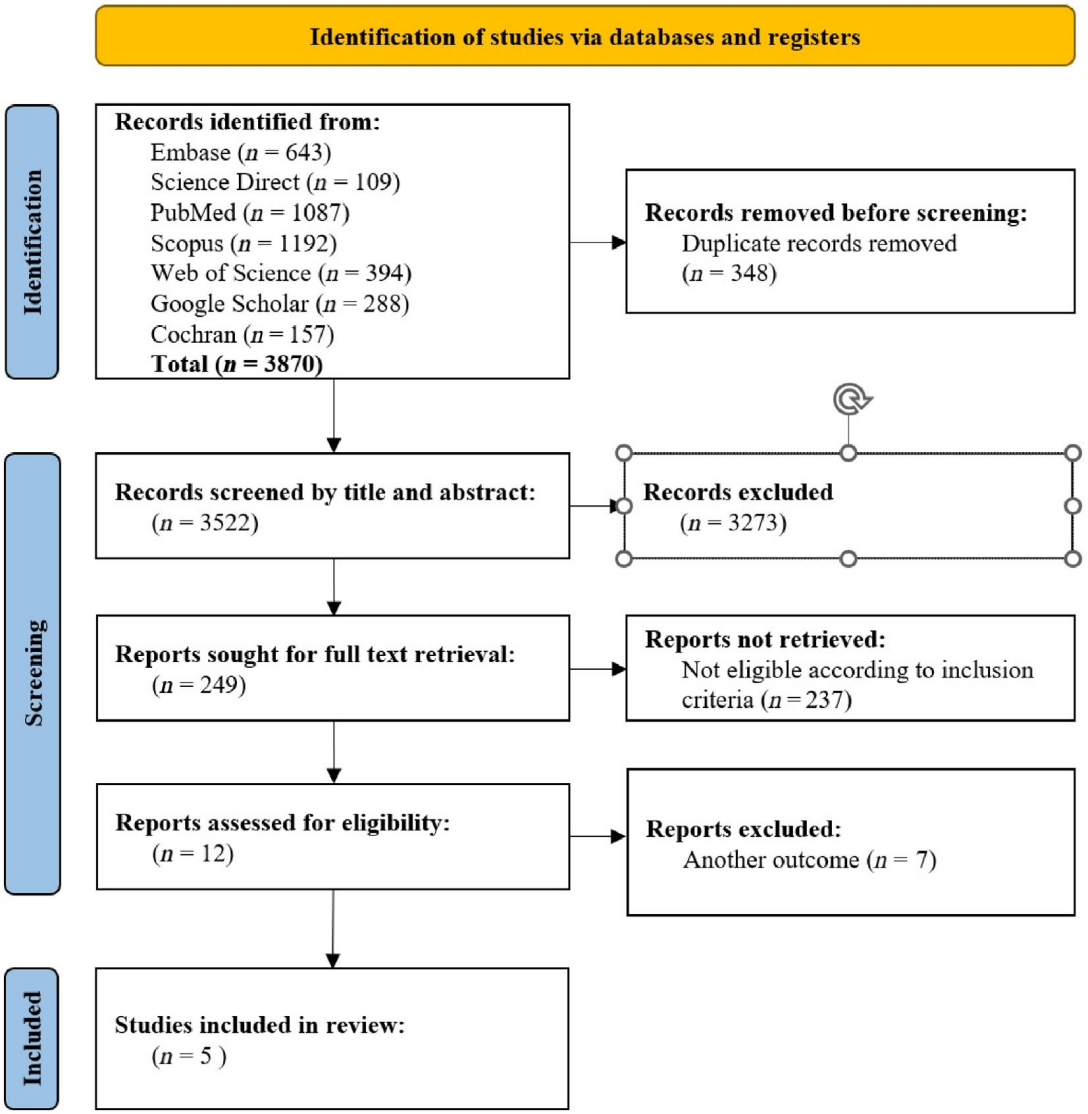
Flowchart of screening process.

Initially, one of the researchers imported all search results from the databases into the EndNote Desktop program, with duplicates removed. Subsequently, two researchers independently reviewed article titles and abstracts based on predetermined eligibility criteria. Any discrepancies in study selection between the two researchers were resolved through a full‐text evaluation. Efforts were made to obtain inaccessible articles and unpublished data by contacting the corresponding authors of eligible studies. Initially, 3870 articles were identified through the database search. Following the elimination of 348 duplicates, 3522 titles and abstracts were screened, leading to the review of 249 full texts. All of them underwent assessment for eligibility criteria culminating in the inclusion of five articles in the study (see Fig. 1). Furthermore, the reference lists of the extracted articles were examined, but no additional articles meeting the inclusion criteria were identified for this study.

Data extraction and synthesis were carried out using a standardised form, which included categories such as study identifiers (study's author, year of publication), country, study objective, study type, parts of materials and methods and outcome (see Table 1).

**Table 1:** Characteristics of included studies

Author(s), year	Country	Objective	Type of study	Inclusion/exclusion criteria	Study population		Study groups		Outcome	Adverse events	Quality assessment
					Participants (*n*)	Participant characteristics	Intervention group	Comparison/control group			
Xue *et al*.^26^	China	Comprehensive geriatric assessment and traditional Chinese medicine intervention benefit symptom control in elderly patients with advanced non‐small cell lung cancer	Randomised trial	**Inclusion criteria:** Histopathologically confirmed advanced non‐small cell lung cancer (NSCLC); American Joint Committee on Cancer (AJCC) stage IIIBIV; C65 years old; capable of reading and understanding the questionnaires; and informed consent. **Exclusion criteria:** Unable to understand the questionnaires; unwilling to participate in this study; and unable to complete the research	**24 elderly patients** with histopathologically proven NSCLC for intervention group 9 non‐elderly NSCLC patients for control group	**Elderly patient:** 13 (54.2%) males and 11 (45.8%) females, mean age was 73.0 ± 5.3 (range 65–83) years, received 65 cycles of chemotherapy in all (mean 2.7 cycles), including paclitaxel, pemetrexed, vinorelbine, or gemcitabine alone or in combination with platinum‐based drug **Non‐elderly patients:** mean age 56.2 ± 4.8 (46–62) years, received 38 cycles of chemotherapy in all (mean 4.2 cycles), including paclitaxel, pemetrexed, vinorelbine, or gemcitabine in combination with platinum‐based drug	Decoctions 150 mL, orally taken on an empty stomach twice daily (every 12 h)	**Control group:** Standardised therapy	Improvement of fatigue symptoms was observed in the intervention group, whereas these symptoms worsened in the control group.	Not mentioned	Moderate
Forbes *et al*.^33^	Unites Kingdom	Physical activity and nutrition interventions for older adults with cancer	Systematic review	**Inclusion criteria:** Lifestyle nutrition and/or activity intervention for people with any cancer diagnosis, measured health‐related quality of life, all participants over 60 years of age and randomised controlled trials. **Exclusion criteria:** Not determined an age range, the intervention was targeting clinicians or carers rather than older adults with cancer, publication language was not in English or findings were conference abstracts only	1660 participants from 14 studies	‐	‐	‐	Nutrition intervention found improvement in depression scores.	Not mentioned	Moderate
Cheng *et al*.^35^	China	Effect of Tai Chi and resistance training on cancer‐related fatigue and quality of life in middle‐aged and elderly cancer patients	Randomised control trial	**Inclusion criteria:** age 55 years old; histopathological diagnosis of lung, gastric and breast cancer; receiving 2–4 courses of chemotherapy and/or radiation therapy; experiencing some degree of cancer‐related fatigue as indicated by the Brief Fatigue Inventory (BFI); willing to be randomised and gave written informed consent to participate. **Exclusion criteria:** Patients with contraindications for RT and TC, such as moderate to severe heart failure, intracranial or bone metastasis, cancerous neuropathy and etc.; mental illness or severe cognitive disorders and/or defects of language expression; regular practice of TC or RT within the past 6 months; and not finished chemotherapy or have discontinued treatment.	120 cancer patients were randomly assigned to 4 groups by a random number table, including TC^a^ group, high‐intensity 60% one repetition maximum (1‐RM) RT group, low‐intensity (30% 1‐RM)^b^ RT group and control group. 105 people were analyzed	‐	**TC group:** 12 weeks of training, 3 times a week for 30 min **RT groups:** 12 weeks with 3 training sessions per week. Each training session consisted of 10 sets, 3 min each	**Control group:** maintaining their original lifestyle and daily life activities	TC has a better effect than RT in terms of sleep quality and mental health. Additionally improved cancer‐related fatigue	There were no adverse events in this trial.	High
Özdemir and Taşcı^27^	Turkey	Acupressure for cancer‐related fatigue in elderly cancer patients	Randomised controlled study	**Inclusion criteria:** The study included patients aged 65 and above who were literate, had completed chemotherapy treatment for cancer at least 1 month prior to the study and had moderate to severe fatigue (visual analogue scale fatigue score 4 and over, scale of 0–10), platelet count above 50 000, haemoglobin levels above 9 g/dL, haematocrit levels above 30%, and patients with estimated survival of more than 3 months. **Exclusion criteria:** Having comorbidities that may cause fatigue such as moderate and severe heart failure, hypothyroidism, diabetes, multiple sclerosis, being diagnosed with a psychiatric disease, having nerve, soft tissue or vascular disease on acupressure areas (hand and leg), having infection or surgical operation on the acupressure areas (hand and leg), and having chemotherapy, radiotherapy or other cancer treatment planned during the study	50 patients randomly divided into two groups: 25 acupressure group, 25 control group. The study was completed with a total of 31 patients (15 acupressure, 16 control group)	20 women (64.5%) and 11 men (35.5%), the education level of most patients is primary (48.4%), most patients are housewives (61.3%), most patients (45.2%)) live with their spouses	Acupressure on three acupuncture points on the hands and legs (LI4, SP6, ST36) by caregivers or the patients themselves for 3 min twice daily, for a period of 4 weeks	No intervention was applied.	Reduction in the severity and level of fatigue	There were no serious side effects.	High
Zhang *et*.*al*.^34^	China	Nutrition and exercise prehabilitation in elderly patients undergoing cancer surgery	Review	‐	‐	‐	‐	‐	Nutritional interventions and counselling can influence psychological indicators, including anxiety and stress responses.	Not mentioned	Moderate

^a^ Tai Chi.

^b^ Resistance training.

### Quality assessment of articles

The Mixed Methods Appraisal Tool (MMAT) was used to evaluate the quality of the studies.^31^ Each section of the tool is categorised based on the research design employed. This tool is valuable for assessing the appropriateness of a study's objective, methods, study design, data collection, study selection, data analysis, presentation of findings, discussion, and conclusion(s). The quality of the articles and their inclusion after data extraction are determined by reviewing these aspects. Articles in each domain are assessed for quality using a percentage scale ranging from 25% (indicating that only one criterion is met) to 100% (indicating that all criteria are met). In this study, articles scoring below 25% are considered low quality, while those scoring above 80% are considered high quality.^31^, ^32^ Based on the findings of the current study, the evaluation of article quality yielded an average score of 75%.

## RESULTS

### Study characteristics

The studies included in this review were conducted in China, the United Kingdom, and Turkey between 2015 and 2023. Among the selected studies, there were three randomised studies and two review studies. The number of elderly cancer patients involved in the randomised studies varied from 24 to 105, with an average age of 65 years. Of the randomised studies, one featured two intervention groups and one control group, while the other two studies each had one intervention group and one control group.

### Types of TCM interventions in improving psychological problems in the elderly with cancer

The five articles presented explored different TCM interventions used to address psychological issues in elderly cancer patients.

One study utilised Chinese herbal decoctions. Patients were prescribed 150 mL of the decoction, taken orally on an empty stomach twice a day (every 12 h). The control group received standard care. The specific ingredients of the decoction were not disclosed in the study.^26^

Two articles focused on nutritional and lifestyle counselling as the primary intervention.^33^, ^34^

Another study employed Tai Chi exercises. Participants performed Tai Chi for 30 min, three times a week, over a 12‐week period. Each session included a 5‐min warm‐up and a 5‐min cool‐down. The study also included two other groups.

The Resistance Training Group engaged in resistance exercises for 12 weeks, with three sessions per week. Each session comprised 10 sets, each lasting 3 min.

The Control Group was instructed to maintain their usual lifestyle and daily activities.^35^

One study investigated the effects of acupressure. Acupressure was applied to three acupuncture points (LI4, SP6, ST36) on the hands and feet. Caregivers or the patients themselves performed acupressure for 3 min, twice a day, over a 4‐week period.^27^

### Psychological consequences of various TCM interventions in the elderly with cancer

The studies reviewed provide evidence that various TCM interventions can significantly improve psychological outcomes in elderly cancer patients. Herbal decoctions, nutritional counselling, Tai Chi and acupressure, can effectively improve various psychological outcomes in elderly cancer patients. These interventions reduce fatigue, depression, anxiety, and stress, while also enhancing sleep quality and overall mental health.

In one study, elderly patients who received herbal decoctions experienced a decrease in fatigue scores, indicating a reduction in fatigue symptoms. Conversely, the control group (non‐elderly patients) saw an increase in fatigue scores, suggesting a worsening of these symptoms.^26^ Another study found that cancer‐related fatigue improved in both the Tai Chi and resistance exercise groups. However, Tai Chi was more effective than resistance training in enhancing sleep quality and mental health.^35^ In a separate study, acupressure significantly reduced the intensity and level of fatigue in elderly cancer patients.^27^ Two studies highlighted the positive effects of nutritional interventions and counselling on psychological indicators. These interventions successfully improved depression scores and had a beneficial impact on anxiety and stress responses, enhancing overall mental well‐being.^33^, ^34^

### Adverse events

In the reviewed studies, the discussion of side effects was incomplete. Three of the articles did not mention side effects at all. One article stated that no side effects were observed.^35^ Another article reported that no serious complications were observed.^27^

## DISCUSSION

This study reviewed the available research with the aim of investigating the effects of TCM on psychological conditions in elderly cancer patients.

The results of the present study showed that herbal decoctions significantly reduce fatigue in elderly cancer patients. TCM methods for managing chronic fatigue have been widely adopted for the treatment of cancer and other conditions in China and elsewhere.^36^, ^37^, ^38^ In oncology care, the most commonly used TCM method in China is a mixture of herbal compounds and decoctions. McCulloch *et al*.^39^ in a multilingual literature review and meta‐analysis, reported that herbal medicine based on *Astragalus mongholicus* (huangqi) was associated with a trend toward increased survival, tumour response, and improved performance status, although these findings were not confirmed due to the quality of the studies reviewed. Additionally, Wisconsin ginseng (*Panax quinquefolius*) was shown to improve cancer‐related fatigue in a phase III double‐blind multicentre study, suggesting that single herbs can be effectively used for managing fatigue in certain patients.^40^

On the other hand, the results indicate that nutritional intervention and counselling effectively alleviate depression, anxiety, and stress responses. The mechanisms through which these dietary changes benefit mental health have yet to be fully established. However, diet may influence mental health through several pathways implicated in mental disorders, including those related to oxidative stress, inflammation, and mitochondrial dysfunction.^41^, ^42^ Gut microbiota dysbiosis has also been implicated, with emerging research demonstrating the involvement of the microbiome in modulating stress response, immune function, neurotransmission, and neurogenesis.^42^ A healthy diet typically contains a wide variety of bioactive compounds that interact beneficially with these pathways. For example, vegetables and fruits not only provide beneficial vitamins, minerals, and fibre but also contain high concentrations of polyphenols, which have been associated with reduced rates of depression in observational studies. This effect is potentially due to their anti‐inflammatory, neuroprotective, and prebiotic properties.^43^ Additionally, vitamins (e.g., B vitamins), fatty acids (e.g., omega‐3 fatty acids), minerals (e.g., zinc, magnesium), and fibre (e.g., resistant starch), as well as other bioactive components (e.g., probiotics), which are typically abundant in healthy dietary patterns, may also protect against mental illness.^42^ Alongside increasing the intake of beneficial nutrients, dietary interventions may enhance mental well‐being by reducing the consumption of unhealthy foods associated with an increased risk of depression, such as processed meats, refined carbohydrates, and other inflammatory foods.^41^

The findings of our study indicate that Tai Chi effectively enhances sleep quality and reduces fatigue in the elderly. Exercise, inflammatory factors, and sleep are interconnected, and Tai Chi, being an aerobic exercise, can improve and regulate inflammatory factors.^44^ These factors play roles not only in immune response and inflammation but also in the neural, endocrine, and immune networks that regulate the sleep wake cycle, thereby enhancing sleep quality.^45^ Research has shown that Tai Chi can increase metabolic rate, improve blood supply to major organs, enhance sleep efficiency, promote deep sleep, and overall improve sleep quality.^46^, ^47^ A meta‐analysis revealed that Tai Chi significantly improves sleep quality and reduces daytime dysfunction, with moderate effects on sleep latency and sleep duration.^48^ Another comprehensive meta‐analysis found that different styles of Tai Chi, including 24‐style, 8‐style, and Yang Tai Chi, have a significant positive impact on sleep quality.^49^

Additionally, the biological mechanisms through which Tai Chi may improve fatigue are not entirely clear.^50^ With respect to the aetiology of treatment‐related fatigue, inflammation, altered immune response, and mitochondrial dysfunction are among the leading hypothesised biological mechanisms.^51^ Tai Chi may have a direct positive impact on immune function and inflammation to reduce fatigue, and it may also indirectly affect these biological pathways through improving cardiorespiratory fitness.^52^

Duan *et al*. found in their meta‐analysis that Tai Chi significantly reduced fatigue in cancer survivors.^53^ Wayne *et al*.'s meta‐analysis revealed that both Tai Chi and Qigong significantly decreased fatigue in breast cancer patients.^54^ Liu *et al*. evaluated two randomised controlled trials (RCTs) involving female breast cancer patients and found that Tai Chi, when used as a supplement to conventional treatments, was more effective in reducing fatigue after 3 months of practice.^55^ Similarly, Song *et al*. found that Tai Chi alleviated fatigue in breast cancer patients and noted that a longer intervention period of 8–12 weeks was more effective in reducing short‐term cancer‐related fatigue compared to shorter durations.^56^

The results of our study showed that acupressure effectively reduced patients' fatigue. Acupressure is a complementary therapy based on TCM's theory of energy pathways. It operates on the premise that restoring proper energy flow through these pathways can enhance health and well‐being, as well as reduce physical symptoms.^57^, ^58^

Studies have indicated that acupressure can help reduce stress, pain, and anxiety.^59^, ^60^ While some studies show that acupressure is not effective in managing fatigue in cancer patients,^61^, ^62^ others report the opposite, suggesting its efficacy in this context.^27^, ^63^, ^64^

A meta‐analysis reported that acupuncture and related treatments are effective in reducing pain and fatigue and enhancing quality of life in cancer patients.^65^ Similarly, a systematic review found that acupuncture and acupressure tend to be effective in relieving fatigue in cancer patients.^66^ Another meta‐analysis specifically highlighted that acupressure is an effective approach for alleviating cancer‐related fatigue.^67^

This study had some limitations. Despite the researchers' attempts to access the full text of all articles, some were not obtained, leading to the exclusion of certain relevant articles from the research. Additionally, while eligible articles were identified and reviewed, some unpublished studies may have been overlooked. The use of specific entry criteria, such as language, could result in the omission of relevant articles. Furthermore, employing a particular search strategy might have led to the exclusion of related articles.

## CONCLUSION

The reviewed studies demonstrate that various TCM interventions, including herbal decoctions, nutritional counselling, Tai Chi, and acupressure, significantly improve psychological outcomes in this population. These interventions exhibit notable efficacy in reducing fatigue, depression, anxiety, and stress levels, while concurrently enhancing sleep quality and overall mental health. However, further research is warranted to validate these findings through RCTs with larger sample sizes. Standardisation of TCM interventions and integration with conventional cancer care are essential steps to optimise patient outcomes. Moreover, patient education on TCM modalities and cultural sensitivity in delivering these interventions are imperative for fostering patient engagement and trust. Long‐term follow‐up studies are needed to assess the sustainability of TCM benefits on psychological well‐being in elderly cancer patients, ensuring comprehensive and holistic care approaches in cancer management.

## AUTHOR CONTRIBUTION

R.Z., P.S., Y.C., W.L, and C.Z. contributed to the study design. Y.C., W.L., and C.Z, supervised data extraction and analysis. R.Z. and P.S. wrote the first draft of the manuscript. All authors critically read and approved the final version of the manuscript.

## DISCLOSURE

The authors declare they have no conflicts of interest in the research.

## Data Availability

The data that support the findings of this study are available from the corresponding author upon reasonable request.

## Appendix I

We developed search strategies tailored to each database search engine to optimise the relevance and precision of our results.

### A.1. Web of Science (Filters*: English, article, proceeding paper, early access)

*TS=(‘Stress* Elderly Cancer*’) OR TS=(‘Anxiety* Elderly Cancer*’) OR TS=(‘Depression* Elderly Cancer*’) OR TS=(‘Post‐Traumatic Disorders* Elderly Cancer*’) OR TS=(‘*R*esilience* Elderly Cancer*’) OR TS=(‘Mental Disorder* Elderly Cancer*’) OR TS=(‘Psychological Disorder* Elderly Cancer*’) OR TS=(‘Psychological Issues* Elderly Cancer*’) OR TS=(‘Sleep Wake Disorders* Elderly Cancer*’) OR TS=(‘Dyssomnias* Elderly Cancer*’) OR TS=(‘Fatigue* Elderly Cancer*’) OR TS=(‘Insomnia* Elderly Cancer*’) OR TS=(‘Traditional Chinese Medicine* Elderly Cancer*’) OR TS=(‘Yin/Yang* Elderly Cancer*’) OR TS=(‘Chinese Decoction* Elderly Cancer*’) OR TS=(‘Chinese Herbal Medicine* Elderly Cancer*’) OR TS=(‘Acupuncture* Elderly Cancer*’) OR TS=(‘Moxibustion* Elderly Cancer*’) OR TS=(‘*Acupressure** Elderly Cancer*’) OR TS=(‘Tui Na Massage* Elderly Cancer*’) OR TS=(‘Cupping* Elderly Cancer*’) OR TS=(‘Nutrition And Lifestyle Consultation* Elderly Cancer*’) OR TS=(‘Meditation* Elderly Cancer*’) OR TS=(‘Taichi* Elderly Cancer*’) TS=(‘Qigong* Elderly Cancer*’)*.

### A.2. Scopus (Filters*: English, article)

*TITLE‐ABS‐KEY(‘Stress* Elderly Cancer*’) OR TITLE‐ABS‐KEY(‘Anxiety* Elderly Cancer*’) OR TITLE‐ABS‐KEY(‘Depression* Elderly Cancer*’) OR TITLE‐ABS‐KEY(‘Post‐Traumatic Disorders* Elderly Cancer*’) OR TITLE‐ABS‐KEY(‘Resilience* Elderly Cancer*’) OR TITLE‐ABS‐KEY(‘Mental Disorder* Elderly Cancer*’) OR TITLE‐ABS‐KEY(‘Psychological Disorder* Elderly Cancer*’) OR TITLE‐ABS‐KEY(‘Psychological Issues* Elderly Cancer*’) TITLE‐ABS‐KEY(‘Sleep Wake Disorders* Elderly Cancer*’) OR TITLE‐ABS‐KEY(‘Dyssomnias* Elderly Cancer*’) OR TITLE‐ABS‐KEY(‘Fatigue* Elderly Cancer*’) OR TITLE‐ABS‐KEY(‘Insomnia* Elderly Cancer*’) OR TITLE‐ABS‐KEY(‘Traditional Chinese Medicine* Elderly Cancer*’) OR TS=(‘Yin/Yang* Elderly Cancer*’) OR TITLE‐ABS‐KEY(‘Chinese Decoction* Elderly Cancer*’) OR TITLE‐ABS‐KEY(‘Chinese Herbal Medicine* Elderly Cancer*’) OR TITLE‐ABS‐KEY (‘Acupuncture* Elderly Cancer*’) OR TITLE‐ABS‐KEY(‘Moxibustion* Elderly Cancer*’) OR TITLE‐ABS‐KEY(‘*Acupressure** Elderly Cancer*’) OR TITLE‐ABS‐KEY(‘Tui Na Massage* Elderly Cancer*’) OR TITLE‐ABS‐KEY(‘Cupping* Elderly Cancer*’) OR TITLE‐ABS‐KEY(‘Nutrition And Lifestyle Consultation* Elderly Cancer*’) OR TITLE‐ABS‐KEY(‘Meditation* Elderly Cancer*’) OR TITLE‐ABS‐KEY(‘Taichi * Elderly Cancer*’) TITLE‐ABS‐KEY(‘Qigong* Elderly Cancer*’)*.

### A.3. PubMed (Filters*: English)

*‘Stress* Elderly Cancer*’[TIAB] OR ‘Anxiety* Elderly Cancer*’[TIAB] OR ‘Depression* Elderly Cancer*’[TIAB] OR ‘Post‐Traumatic Disorders* Elderly Cancer*’[TIAB] OR ‘*R*esilience* Elderly Cancer*’[TIAB] OR ‘Mental Disorder* Elderly Cancer*’[TIAB] OR ‘Psychological Disorder* Elderly Cancer*’[TIAB] OR ‘Psychological Issues* Elderly Cancer*’[TIAB] OR ‘Sleep Wake Disorders* Elderly Cancer*’[TIAB] OR ‘Dyssomnias* Elderly Cancer*’[TIAB] OR ‘Fatigue* Elderly Cancer*’[TIAB] OR ‘Insomnia* Elderly Cancer*’[TIAB] OR ‘Traditional Chinese Medicine* Elderly Cancer*’[TIAB] OR ‘Yin/Yang* Elderly Cancer*’[TIAB] OR ‘Chinese Decoction* Elderly Cancer*’[TIAB] OR ‘Chinese Herbal Medicine* Elderly Cancer*’[TIAB] OR ‘Acupuncture* Elderly Cancer*’[TIAB] OR ‘Moxibustion* Elderly Cancer*’[TIAB] OR ‘*Acupressure** Elderly Cancer*’[TIAB] OR ‘Tui Na Massage* Elderly Cancer*’[TIAB] OR ‘Cupping* Elderly Cancer*’[TIAB] OR ‘Nutrition And Lifestyle Consultation* Elderly Cancer*’[TIAB] OR ‘Meditation* Elderly Cancer*’[TIAB] OR ‘Taichi * Elderly Cancer*’[TIAB] ‘Qigong* Elderly Cancer*’[TIAB]*.

### A.4. EMBASE (Filters*: English, article, article in press, preprint)

*‘Stress* Elderly Cancer*’:ti, ab, kw OR ‘Anxiety* Elderly Cancer*’:ti, ab, kw OR ‘Depression* Elderly Cancer*’:ti, ab, kw OR ‘Post‐Traumatic Disorders* Elderly Cancer*’:ti, ab, kw OR ‘*R*esilience* Elderly Cancer*’:ti, ab, kw OR ‘Mental Disorder* Elderly Cancer*’:ti, ab, kw OR ‘Psychological Disorder* Elderly Cancer*’:ti, ab, kw OR ‘Psychological Issues* Elderly Cancer*’:ti, ab, kw OR ‘Sleep Wake Disorders* Elderly Cancer*’:ti, ab, kw OR ‘Dyssomnias* Elderly Cancer*’:ti, ab, kw OR ‘Fatigue* Elderly Cancer*’:ti, ab, kw OR ‘Insomnia* Elderly Cancer*’:ti, ab, kw OR ‘Traditional Chinese Medicine* Elderly Cancer*’:ti, ab, kw OR ‘Yin/Yang* Elderly Cancer*’:ti, ab, kw OR ‘Chinese Decoction* Elderly Cancer*’:ti, ab, kw OR ‘Chinese Herbal Medicine* Elderly Cancer*’:ti, ab, kw OR ‘Acupuncture* Elderly Cancer*’:ti, ab, kw OR ‘Moxibustion* Elderly Cancer*’:ti, ab, kw OR ‘Acupressure* Elderly Cancer*:ti, ab, kw OR ‘Tui Na Massage* Elderly Cancer*’:ti, ab, kw OR ‘Cupping* Elderly Cancer*’:ti, ab, kw OR ‘Nutrition And Lifestyle Consultation* Elderly Cancer*’:ti, ab, kw OR ‘Meditation* Elderly Cancer*’:ti, ab, kw OR ‘Taichi * Elderly Cancer*’:ti, ab, kw ‘Qigong* Elderly Cancer*’:ti, ab, kw*.

### A.5. Google Scholar (Filters: none)

*allintitle: Stress Elderly Cancer*
*allintitle: Anxiety Elderly Cancer*
*allintitle: Depression Elderly Cancer*
*allintitle: Post‐Traumatic Disorders Elderly Cancer*
*allintitle: Resilience Elderly Cancer*
*allintitle: Mental Disorder Elderly Cancer*
*allintitle: Psychological Disorder Elderly Cancer*
*allintitle: Psychological Issues Elderly Cancer*
*allintitle: Sleep Wake Disorders Elderly Cance*
*allintitle: Dyssomnias Elderly Cancer*
*allintitle: Fatigue Elderly Cancer*
*allintitle: Insomnia Elderly Cancer*
*allintitle: Traditional Chinese Medicine Elderly Cancer*
*allintitle: Yin/Yang Elderly Cancer*
*allintitle: Chinese Decoction Elderly Cancer*
*allintitle: Chinese Herbal Medicine Elderly Cancer*
*allintitle: Acupuncture Elderly Cancer*
*allintitle: Moxibustion Elderly Cancer*
*allintitle: Acupressure Elderly Cancer*
*allintitle: Tui Na Massage Elderly Cancer*
*allintitle: Cupping Elderly Cancer*
*allintitle: Nutrition And Lifestyle Consultation Elderly Cancer*
*allintitle: Meditation Elderly Cancer*
*allintitle: Taichi Elderly Cancer*
*allintitle: Qigong Elderly Cancer*

*After retrieving search results, filters were applied using the filter panel on the results page.
